# Association between gaseous pollutants and emergency ambulance dispatches for asthma in Chengdu, China: a time-stratified case-crossover study

**DOI:** 10.1186/s12199-019-0773-0

**Published:** 2019-03-18

**Authors:** Jianyu Chen, Xianyan Jiang, Chunli Shi, Ruicong Liu, Rong Lu, Li Zhang

**Affiliations:** 1 Sichuan Provincial Center for Disease Control and Prevention, No.6, Zhongxue Road, Wuhou District, Chengdu, 610041 China; 2 Chengdu Center for Disease Control and Prevention, Chengdu, China

**Keywords:** Air pollution, Basin, Emergency ambulance dispatches, Asthma, Time-stratified case-crossover

## Abstract

**Objectives:**

The association between concentrations of sulfur dioxide (SO_2_), nitrogen dioxide (NO_2_), carbon monoxide (CO), ozone (O_3_), and emergency ambulance dispatches (EADs) for asthma was explored in the central Sichuan Basin of southwestern China for the first time.

**Methods:**

EADs for asthma were collected from the Chengdu First-Aid Command Center. Pollutant concentrations were collected from 24 municipal environmental monitoring centers and including SO_2_, NO_2_, CO, daily 8-h mean concentrations of O_3_ (O_3_-8 h), and particulate matter less than 2.5 μm in aerodynamic diameter (PM_2.5_). The climatic data were collected from the Chengdu Municipal Meteorological Bureau. All data were collected from years spanning 2013–2017. A time-stratified case-crossover design was used to analyze the data.

**Results:**

After controlling for temperature, relative humidity, and atmospheric pressure, IQR increases in SO_2_ (13 μg/m^3^), NO_2_ (17 μg/m^3^), and CO (498 μg/m^3^) were associated with 18.8%, 11.5%, and 3.1% increases in EADs for asthma, respectively. The associations were strongest for EADs and SO_2_, NO_2_, and CO levels with 3-, 5-, and 1-day lags, respectively.

**Conclusions:**

This study provides additional data to the limited body of literature for potential health risks arising from ambient gaseous pollutants. The results of the study suggest that increased concentrations of SO_2_, NO_2_, and CO were positively associated with emergency ambulance dispatches for asthma in Chengdu, China. Further studies are needed to investigate the effects of individual air pollutants on asthma.

## Introduction

Asthma is one of the most common chronic diseases globally. As many as 300 million people currently have asthma, and it accounts for about 1 in 250 deaths worldwide. Moreover, the prevalence and patient burden from asthma have been sharply increasing [1]. Estimates for the costs of asthma in six developed countries range from $300 to $1300 per patient per year adjusted to 1990 US dollars [2]. Additional analyses indicate that asthma is estimated to cost 1–2% of the total health care budget of developed economies. In particular, asthma prevalence is sharply rising in developing countries and regions with increasing urbanization and westernization when compared to developed countries.

Previous studies have demonstrated the relationship between ambient air pollutants and asthma [3–6]. For example, associations have been detected between ambient air pollutant concentrations and the rate of pediatric asthma emergency department visits in Atlanta, USA [7]. Furthermore, Cai et al. (2014) reported that sulfur dioxide (SO_2_) and nitrogen dioxide (NO_2_) levels are more associated with asthma hospitalization events in Shanghai, China compared to levels of particulate matter with aerodynamic diameters less than 10 μm (PM_10_) [3]. The effects of air pollutants on asthma prevalence can also be associated with specific geographic terrains, weather conditions, and ethnic populations [8–11]. Following these previous analyses, the goal of the present study was to evaluate the association between short-term exposure to ambient gaseous pollutants (SO_2_, NO_2_, carbon monoxide (CO), and ozone (O_3_)) and emergency ambulance dispatches (EADs) for asthma in a typical basin of southwestern China.

The prevalence of gaseous pollutants including SO_2_, NO_2_, CO, and O_3_ were evaluated in comparison with asthma prevalence in the Sichuan Basin. The Sichuan Basin is a typical basin of southwestern China and exhibits severe air pollution [12]. To our knowledge, this report is the first to investigate the association between gaseous pollutants and asthma in this region, and thus, represents an important contribution to understanding the relationship between pollution and asthma for this region, and in general. The city of Chengdu was used as the study area, which comprises a population of over 15 million people [13]. Chengdu features its own unique geographical and meteorological conditions that are due to its location at the central region of the Sichuan Basin. EADs for asthma cases were investigated that encompassed cases across the whole city owing to data from the city’s Municipal First-Aid Command System. The association between air pollutants and EADs for asthma has rarely been explored in previous studies. However, some studies have explored the associations between air pollutants and EADs for cardiovascular diseases, respiratory diseases, and other systemic diseases [14, 15]. Consequently, we conducted a time-stratified case-crossover experimental design to explore the association between ambient gaseous pollutants and EADs for asthma in the central Sichuan Basin of China for the first time.

## Materials and methods

### Data collection

Data for EADs from asthma were collected from the Chengdu First-Aid Command Center from 1 January 2013 to 31 December 2017. The Chengdu First-Aid Command Center responds to all emergency calls for first aid in Chengdu, dispatches ambulances to transport patients to emergency hospitals, and serves residents of the entire city. EAD records include the patient identification number, gender, age, dispatch date, patient address, address code, chief complaint, and primary diagnosis. When a patient conducts an emergency call (number 120), their primary information including gender, age, and chief complaint is queried and recorded by operators at the Chengdu First-Aid Command Center. Patient identification numbers and dispatch dates are concomitantly generated automatically by the system. Primary diagnoses for patients are obtained by return calling the emergency hospital where the patient was dispatched immediately following the diagnosis. All case data from 1 January 2013 to 31 December 2017 were initially collected, and then only those with a primary diagnosis of asthma were used further.

**Fig. 1:**
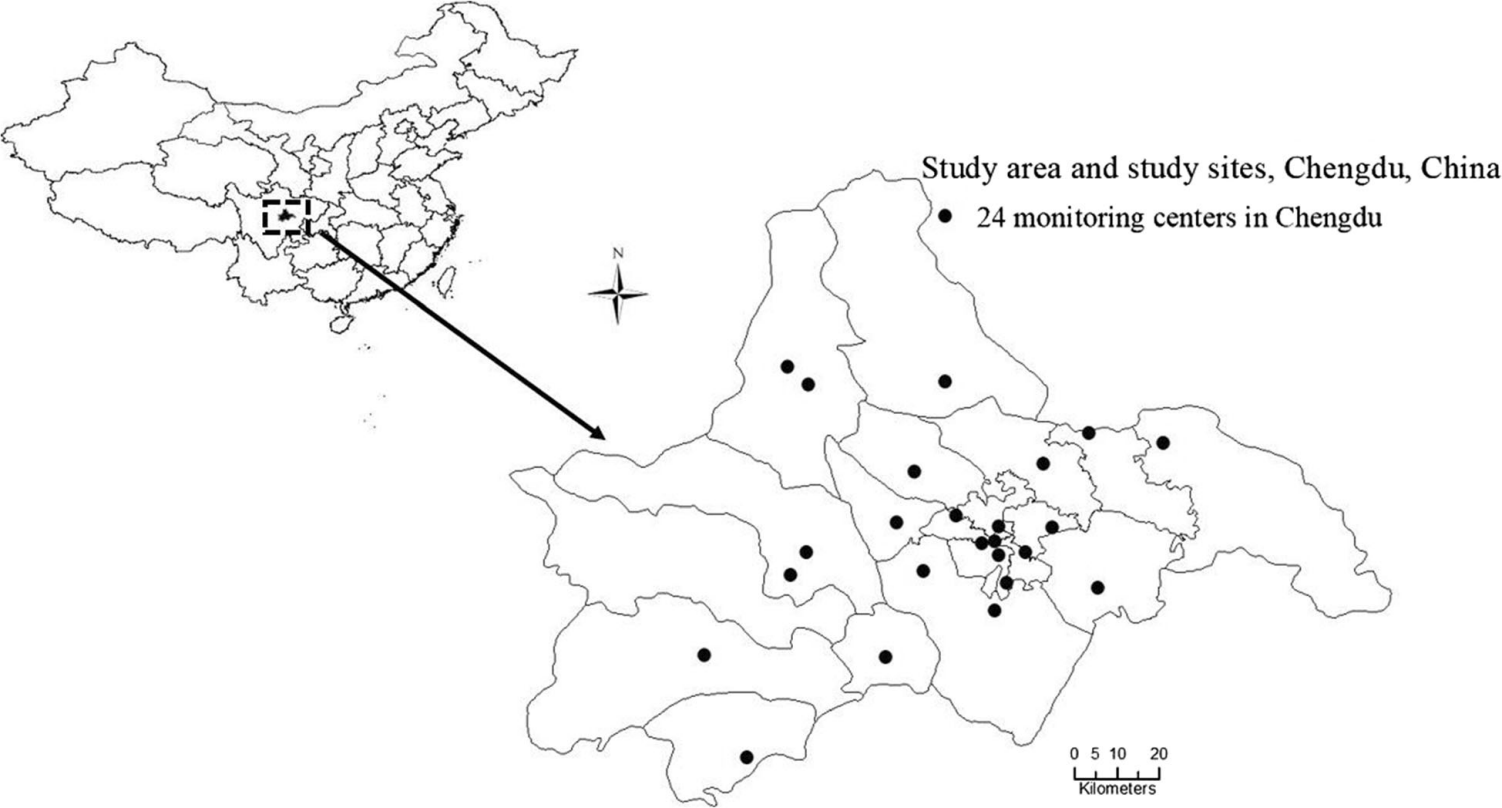
Study area and study locations within the city of Chengdu, China. The enlarged area depicts the spatial distribution of the 24 municipal environmental monitoring centers throughout Chengdu

Air pollutant concentrations were collected from all 24 Municipal Environmental Monitoring Centers in Chengdu from 1 January 2013 to 31 December 2017 (Fig. 1) and comprised data for SO_2_, NO_2_, CO, daily 8-h mean concentrations of O_3_ (O_3_-8 h), and particulate matter with aerodynamic diameters less than 2.5 μm (PM_2.5_). Daily mean concentrations for SO_2_, NO_2_, CO, O_3_-8 h, and PM_2.5_ were calculated using data for each pollutant from all 24 centers. Daily 8-h mean concentrations of O_3_ were defined as the highest 8-h mean concentration for O_3_ per day. If data from one or more centers were missing for a given day, data from the other centers would be used to calculate the mean concentrations that individuals were exposed to. Numerous previous analyses have considered weather conditions as confounding factors that should be adequately controlled for [16, 17]. Therefore, climatic parameters were collected from the Chengdu Municipal Meteorological Bureau from 1 January 2013 to 31 December 2017 that included daily mean temperature, relative humidity, and atmospheric pressure.

### Statistic analyses

Spearman’s correlational analyses were used to explore correlations between air pollutants and weather conditions. To estimate the association between gaseous pollutants and EADs for asthma, a time-stratified case-crossover methodology was used. The case-crossover method restricts the controls to the cases themselves. Consequently, the influence of confounding variables related to individual characteristics like gender, age, occupational hazards, smoking, and basic disease could be adequately controlled [18]. The R package “season” was used, allowing alternative values of controls to be set in the model. Time stratification was conducted to control for confounding variables due to long-term trends, seasonal patterns, and the day of the week [19]. Controls were restricted to the same weekday within the same month and year, relative to the corresponding cases. By controlling in this manner, the influences of trends due to time (long-term trends and seasonal patterns) and the day of the week are mitigated. The number of controls that were used was varied from three to four according to the number of days in a month. For example, if a case was dispatched to an emergency hospital on Friday, 23 September 2016, the case day was defined as the same day, and control days were assigned as all of the remaining Fridays in September 2016 (i.e., the 2nd, 9th, 16th, and 30th).

Delayed effects might exist between gaseous pollutant exposure and health outcomes [20–22]. To assess the potential for delayed effects on EAD instances for asthma, a single-day lag model for each gaseous pollutant was conducted by ranging the lag days from one to seven.

The influence of confounding variables including daily mean temperature, relative humidity, and atmospheric pressure was controlled by using natural cubic splines (ns) in the R software environment with 3° of freedom for smoothing in all models [23].

Sensitivity analyses were conducted in several manners to assess the robustness of the results. First, the setting of controls was varied. Three controls were restricted to one case in the models, while ignoring if cases and controls originated from the same month [24, 25]. Second, a two-pollutant model was assessed for each pollutant. Third, a multi-day moving average was calculated for pollutant concentrations extending from the day of analysis and up to 3 days prior (lag 01, lag 02, and lag 03, respectively). The concentrations for each pollutant from the day of EAD to 1-, 2-, and 3-days prior were used to calculate moving average concentrations to approximate exposures, rather than calculating mean concentrations for each pollutant on each day.

Model results are presented as odds ratios (ORs) with 95% confidence intervals (95% CI) with increased concentrations of each pollutant given per interquartile range (IQR) [25, 26]. The season package for R (version 3.5.1) was used for fitting the time-stratified case-crossover model [27].

## Results

A total of 2669 EADs for asthma were observed in Chengdu between 1 January 2013 and 31 December 2017. The mean concentrations of SO_2_, NO_2_, CO, O_3_-8 h, and PM_2.5_ were 21.6, 41.5, 1124.2, 92.4, and 70.5 μg/m^3^, respectively, while increases in their IQRs were 13, 17, 498, 74, and 53 μg/m^3^, respectively. The values for mean daily temperature, relative humidity, and atmospheric pressure were 17.0 °C, 77.7%, and 951.7 hpa, respectively (Table 1).

**Table 1:** Air pollutant, weather conditions, and emergency ambulance dispatches for asthma cases between 2013 and 2017 in Chengdu

	Mean	SD	Min.	25%	50%	75%	Max.	IQR
SO_2_ (μg/m^3^)	21.6	10.5	6.0	14.0	20.0	27.0	71.0	13.0
NO_2_ (μg/m^3^)	41.5	13.2	13.0	32.0	39.0	49.0	89.0	17.0
CO (μg/m^3^)	1124.2	790.2	425.0	787.0	987.0	1285.0	16,608.0	498.0
O_3_-8 h (μg/m^3^)	92.4	49.6	11.0	54.0	82.0	128.0	285.0	74.0
PM_2.5_ (μg/m^3^)	70.5	48.9	9.0	36.0	56.0	89.0	372.0	53.0
Temperature (°C)	17.0	7.2	−1.9	10.3	17.9	23.2	30.0	12.9
Humidity (%)	77.7	9.7	41.0	72.0	78.0	85.0	98.0	13.0
AP (hpa)	951.7	7.4	933.1	945.6	951.2	957.4	977.3	11.8
EADs (tpd)	1.5	1.3	0.0	0.0	1.0	2.0	6.0	2.0

*Abbreviations*: *SO*_*2*_ sulfur dioxide, *NO*_*2*_ nitrogen dioxide, *CO* carbon monoxide, *O*_*3*_*-8 h* daily 8-h mean concentration of O_3_, *PM*_*2.5*_ particulate matter less than 2.5 μm in aerodynamic diameter, *AP* atmospheric pressure, *EADs* emergency ambulance dispatches, *tpd* times per day, *SD* standard deviation, *IQR* interquartile range

**Fig. 2:**
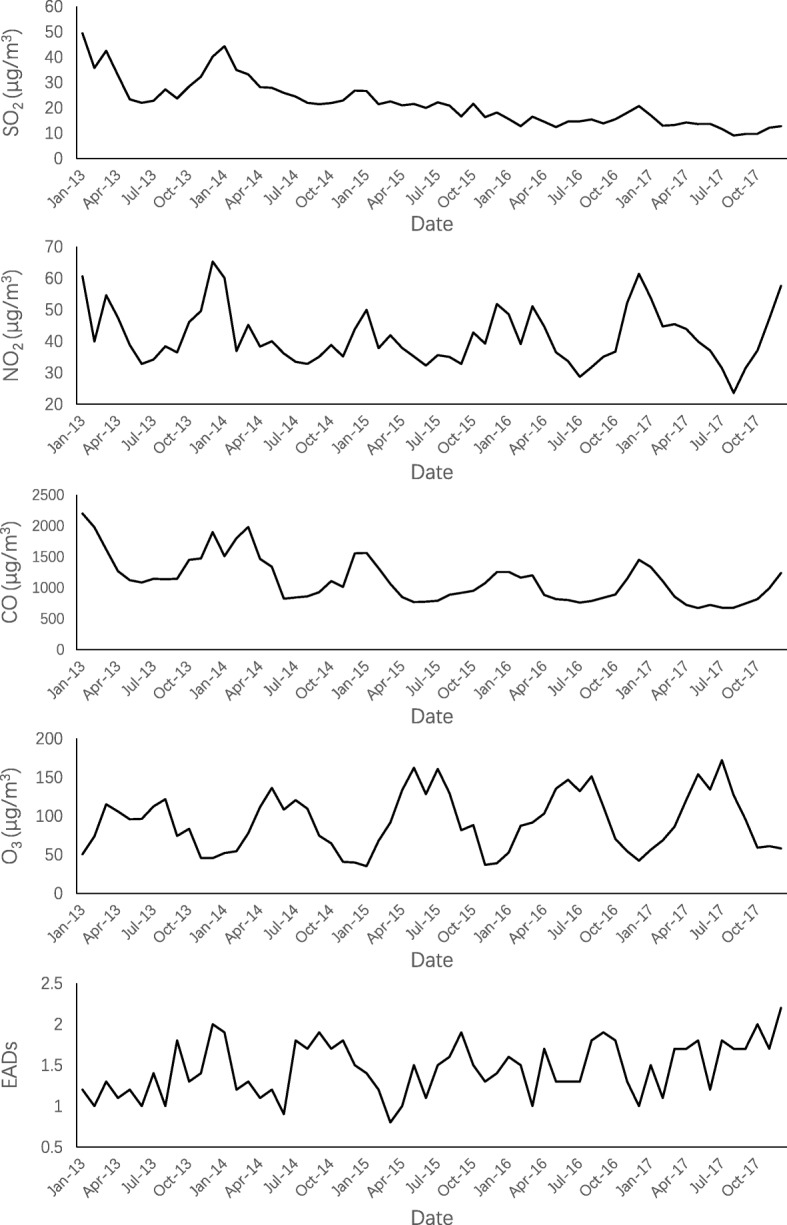
Seasonal trends of monthly mean concentrations of SO_2_, NO_2_, CO, and O_3_, and EADs

Spearman’s correlation analysis indicated the presence of correlations among several air pollutants, including SO_2_ and NO_2_ (*r* = 0.474, *P* < 0.05), SO_2_ and CO (*r* = 0.615, *P* < 0.05), SO_2_ and O_3_-8 h (*r* = − 0.047, *P* < 0.05), SO_2_ and PM_2.5_ (*r* = 0.622, *P* < 0.05), NO_2_ and CO (*r* = 0.678, *P* < 0.05), NO_2_ and O_3_-8 h (*r* = − 0.195, *P* < 0.05), NO_2_ and PM_2.5_ (*r* = 0.785, *P* < 0.05), CO and O_3_-8 h (*r* = − 0.383, *P* < 0.05), CO and PM_2.5_ (*r* = 0.817, *P* < 0.05), and O_3_-8 h and PM_2.5_ (*r* = − 0.205, *P* < 0.05). In addition, correlations were observed between air pollutants and weather conditions (Table 2). The concentrations of SO_2_, NO_2_, and CO were remarkably higher during winter than during summer, while O_3_ concentrations were highest in the summers of each year. The EADs were also relatively higher during winters (Fig. 2).

**Table 2:** Spearman’s correlation coefficients for relationships between air pollutants and weather conditions

	SO_2_	NO_2_	CO	O_3_	PM_2.5_	Temperature	Humidity	AP
SO_2_	1							
NO_2_	0.474^a^	1						
CO	0.615^a^	0.678 ^a^	1					
O_3_-8 h	− 0.047^a^	− 0.195^a^	− 0.383^a^	1				
PM_2.5_	0.622^a^	0.785^a^	0.817^a^	− 0.205^a^	1			
Temperature	− 0.084^a^	− 0.431^a^	− 0.474^a^	0.680^a^	− 0.462^a^	1		
Humidity	− 0.446^a^	− 0.137^a^	− 0.058^a^	− 0.428^a^	− 0.190^a^	− 0.078^a^	1	
AP	0.079^a^	0.365^a^	0.363^a^	− 0.596^a^	0.335^a^	− 0.851^a^	− 0.002	1

*Abbreviations*: *SO*_*2*_ sulfur dioxide, *NO*_*2*_ nitrogen dioxide, *CO* carbon monoxide, *O*_*3*_*-8 h* daily 8-h mean concentration of O_3_, *PM*_*2.5*_ particulate matter less than 2.5 μm in aerodynamic diameter, *AP* atmospheric pressure

^a^*P* < 0.05

**Fig. 3:**
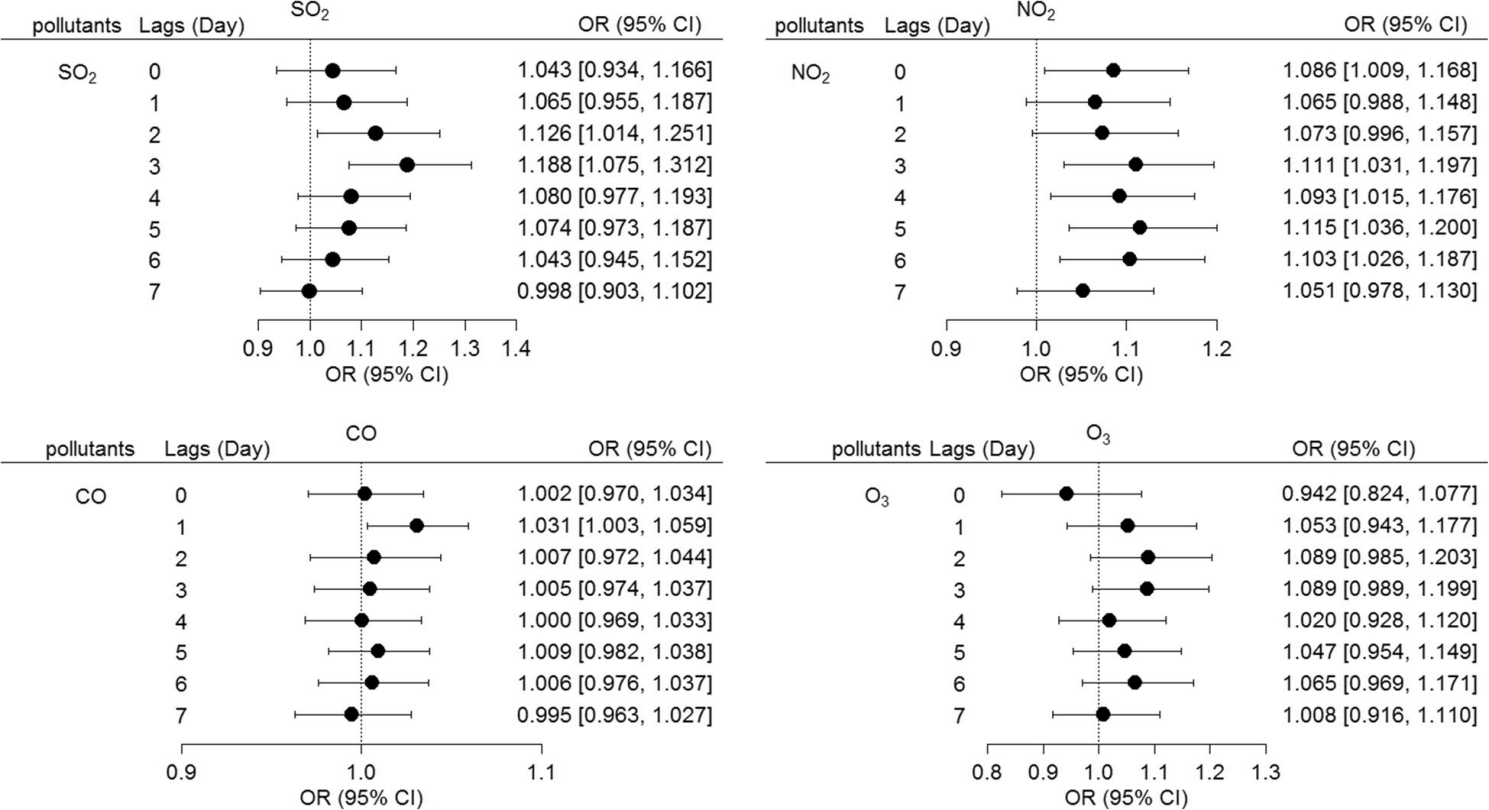
Association between EADs for asthma and IQR increases for SO_2_, NO_2,_ CO, and O_3_ over lag 0 to lag 7 days. All models were adjusted for temperature, relative humidity, and atmospheric pressure

After controlling for temperature, relative humidity, and atmospheric pressure, an IQR increase (13 μg/m^3^) of SO_2_ was significantly and positively associated with EADs for asthma at lag 2 day (OR = 1.126, 95% CI 1.014–1.251) and lag 3 day (OR = 1.188, 95% CI 1.075–1.312). In addition, an IQR increase (17 μg/m^3^) for NO_2_ was significantly and positively associated with EADs for asthma at lag 0 day (OR = 1.086, 95% CI 1.009–1.168), lag 3 day (OR = 1.111, 95% CI 1.031–1.197), lag 4 day (OR = 1.093, 95% CI 1.015–1.176), lag 5 day (OR = 1.115, 95% CI 1.036–1.200), and lag 6 day (OR = 1.103, 95% CI 1.026–1.187). Finally, an IQR increase (498 μg/m^3^) of CO was significantly and positively associated with EADs for asthma at lag 1 day (OR = 1.031, 95% CI 1.003–1.059) (Fig. 3). The days corresponding to the greatest effects due to SO_2_, NO_2_, and CO were 3, 5, and 1 day lags, respectively.

In the two-pollutant models, there was an association between cases and SO_2_ at lag 3 day after adjusting for PM_2.5_ (OR = 1.204, 95% CI 1.058–1.371), NO_2_ (OR = 1.157, 95% CI 1.004–1.333), CO (OR = 1.193, 95% CI 1.077–1.321), and O_3_ (OR = 1.174, 95% CI 1.060–1.301). There was also an observed association between cases and NO_2_ at lag 5 day after adjusting for SO_2_ (OR = 1.158, 95% CI 1.042–1.288), CO (OR = 1.115, 95% CI 1.034–1.202), and O_3_ (OR = 1.111, 95% CI 1.031–1.197). Lastly, an association was observed for CO at lag 1 day after adjusting for SO_2_ (OR = 1.029, 95% CI 1.001–1.058) and O_3_ (OR = 1.032, 95% CI 1.004–1.060) (Table 3).

**Table 3:** Odds ratios (OR) for two-pollutant models including SO_2_, NO_2_, and CO^b^

Air pollutants		OR	OR 95% CI	
			Lower bound	Upper bound
SO_2_		1.188^a^	1.075	1.312
	+PM_2.5_	1.204^a^	1.058	1.371
	+NO_2_	1.157^a^	1.004	1.333
	+CO	1.193^a^	1.077	1.321
	+O_3_	1.174^a^	1.060	1.301
NO_2_		1.115^a^	1.036	1.200
	+PM_2.5_	1.068	0.960	1.189
	+SO_2_	1.158^a^	1.042	1.288
	+CO	1.115^a^	1.034	1.202
	+O_3_	1.111^a^	1.031	1.197
CO		1.031^a^	1.003	1.059
	+PM_2.5_	1.028	1.000	1.057
	+SO_2_	1.029^a^	1.001	1.058
	+NO_2_	1.027	0.999	1.056
	+O_3_	1.032^a^	1.004	1.060

*Abbreviations*: *SO*_*2*_ sulfur dioxide, *NO*_*2*_ nitrogen dioxide, *CO* carbon monoxide, *OR* odds ratio, *CI* confidence interval

^a^*P* < 0.05

^b^ORs for SO_2_, NO_2_, and CO are derived from 3-, 5-, and 1-day lags, respectively

**Fig. 4:**
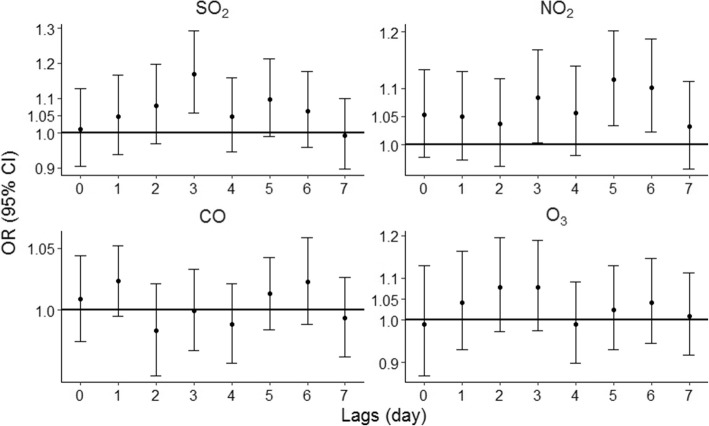
Association between EADs for asthma and IQR increases in SO_2_, NO_2,_ CO, and O_3_ levels with lag 0 to lag 7 days. The results derive from controlled analyses after altering the lag structure for each pollutant by restricting three fixed controls to one case

Restricting three fixed controls to one case instead of restricting cases and controls to being from the same month resulted in similar values and trends of ORs for each pollutant (Fig. 4). In addition, ORs calculated via different lag-day structures (lag 01, lag 02, and lag 03, respectively) were similar to those from the single-day lag models (Table 4). Thus, these sensitivity analyses suggest that the results of the models were reliable.

**Table 4:** Odds ratios for different lag-day model structures of air pollutants

Air pollutants	Lag (days)	OR	OR 95% CI	
			Lower bound	Upper bound
SO_2_	0	1.043	0.934	1.166
	1	1.065	0.955	1.187
	2	1.126^a^	1.014	1.251
	3	1.188^a^	1.075	1.312
	01	1.072	0.945	1.216
	02	1.131	0.986	1.298
	03	1.219^a^	1.053	1.412
NO_2_	0	1.086^a^	1.009	1.168
	1	1.065	0.988	1.148
	2	1.073	0.996	1.157
	3	1.111^a^	1.031	1.197
	01	1.093^a^	1.007	1.187
	02	1.111^a^	1.015	1.215
	03	1.146^a^	1.040	1.263
CO	0	1.002	0.970	1.034
	1	1.031^a^	1.003	1.059
	2	1.007	0.972	1.044
	3	1.005	0.974	1.037
	01	1.030	0.992	1.071
	02	1.036	0.988	1.088
	03	1.038	0.983	1.096
O_3_	0	0.942	0.824	1.077
	1	1.053	0.943	1.177
	2	1.089	0.985	1.203
	3	1.089	0.989	1.199
	01	1.011	0.865	1.181
	02	1.087	0.923	1.280
	03	1.141	0.964	1.350

*Abbreviations*: *SO*_*2*_ sulfur dioxide, *NO*_*2*_ nitrogen dioxide, *CO* carbon monoxide, *O*_*3*_ ozone, *OR* odds ratio, *CI* confidence interval

^a^*P* < 0.05

## Discussion

A significantly positive association was observed between EADs and gaseous pollutants when using a time-stratified case-crossover methodology. After controlling for temperature, relative humidity, and atmospheric pressure, IQR increases in SO_2_ (13 μg/m^3^), NO_2_ (17 μg/m^3^), and CO (498 μg/m^3^) were associated with 18.8%, 11.5%, and 3.1% increases in EADs for asthma, respectively. These effects were most notable for SO_2_, NO_2_, and CO at 3-, 5-, and 1-day lags, respectively. Furthermore, associations were observed between EADs for asthma and SO_2_ and NO_2_ when using lag models with other days of lag. Delay effects were only observed between asthma and exposure to SO2, CO, and EADs, but not between asthma incidences and NO_2_. Moreover, the health impacts of NO_2_ exposure as measured by EADS for asthma were similar among 3- to 6–day lag windows. IQR increases for NO_2_ at 3-, 4-, 5-, and 6-day lags were associated with 11.1%, 9.3%, 11.5%, and 10.3% increases in EADs for asthma, respectively, indicating that there may be a plateauing lag period for the association between NO_2_ and EADS for asthma. Several studies have suggested that further investigations are needed to evaluate the association between O_3_ and asthma in the Sichuan Basin [28, 29]. However, an association between exposure to O_3_ and EADs for asthma was not observed in our analyses. The major sources of SO_2_, NO_2_, CO, and O_3_ pollutants in Chengdu arise from industrial emissions and fuel combustion. In addition, traffic emissions also contribute to ambient NO_2_ and CO levels [30, 31]. The concentrations of gaseous pollutants exhibited typical seasonal trends. Specifically, the concentrations of SO_2_, NO_2_, and CO were remarkably higher during winters, which may be due to reduced dilution efficiency via air flow diffusion, and increases in biomass fuel combustion for heating. In addition, higher O_3_ concentrations in summer were likely caused by increased sunlight. Nevertheless, the aforementioned seasonal trends were accounted for in our analyses by using the case-crossover method.

The results reported here are consistent with those from previous studies. Ding et al. [32] explored the association between air pollution and hospital visits for asthma attack in children of Chongqing, China using a case-crossover analysis. The analysis indicated that increases of 10 μg/m^3^ concentrations in SO_2_, NO_2_, and CO were associated with 4.2%, 9.0%, and 0.4% increases in hospital visits for asthma attacks in children [32]. Furthermore, an analysis of several cities in the USA, including Atlanta, Dallas, and St. Louis [33], revealed associations between pollutant concentrations and asthma emergency department visits. In particular, the associations were strongest for patients in the 5–18-year age group, wherein the ratios per IQR (5 p.p.b. for SO_2_, 12 p.p.b. for NO_2_, 0.5 p.p.m. for CO, and 28 p.p.b. for O_3_) were 1.01%, 1.05%, 1.03, and 1.07%, respectively. Similarly, a cohort study in Denmark comprising 53,695 people [34] indicated that NO_2_ levels were associated with increased risk of hospitalization for asthma, with an adjusted hazard ratio per IQR (5.8 μg/m^3^) of 1.12. Likewise, a cohort study of 5349 children attending kindergarten and first grade in Southern California [35] revealed associations between new-onset asthma and NO_2_ levels.

The lag days exhibiting the greatest effect for each pollutant were used for two-pollutant models. Modeling revealed that ORs for NO_2_ decreased after adjusting for PM_2.5_, and a similar decrease was observed for CO after adjusting for PM_2.5_, and NO_2_. These results are consistent with those from previous studies [32], indicating the presence of synergistic effects among air pollutants towards disease incidence.

SO_2_ has long been considered an environmental cause of asthma. For example, Romanoff discussed the toxic effects of SO_2_ fume inhalation on bronchial asthma in 1939 [36]. One possible mechanism underlying aggravation of asthma by SO_2_ could be that SO_2_ derivatives increase the expression levels of EGF, EGFR, ICAM-1, and COX-2 proteins in BEP2D cells. The upregulation of these proteins then results in mucus over-production and inflammatory responses [37]. An experiment with newborn Sprague–Dawley rats sensitized to ovalbumin revealed that SO_2_ could be a universal factor in airway inflammatory processes that specifically exacerbates airway hyperresponsiveness (AHR) in asthmatics [38]. The underlying mechanism of this response could be due to increases in Penh (an indicator of AHR), that then increases antibody IL-4 production in serum and increases airway smooth muscle cell stiffness and contractility.

Exposure to ambient NO_2_ may enhance bronchial hypersensitivity to asthma, promote allergen sensitization to inhaled antigens, and increase the risk of exacerbating asthma following respiratory infections [39–42]. Mechanisms underlying the exacerbation of asthma by NO_2_ that may be of particular significance include the following: (1) enhancing induction of apoptosis in normal human bronchial epithelial cells and inducing cell damage [43]. 2) Inducing an upregulation of IL-5, IL-10, IL-13, and ICAM-1 antibodies in the bronchial epithelium, thereby exerting a “pro-allergic” effect [44].

A relationship between ambient CO levels and asthma incidence has been demonstrated in many previous studies [7, 32, 33, 45, 46]. However, the biological mechanism underlying this association has not yet been determined. Norris et al. (1999) considered CO as a general indicator for the effects of air pollution [47]. In contrast to the above studies, recent studies have suggested that CO may potentially play a protective role in respiratory systems [48]. Ameredes et al. (2003) reported that exposure to CO could reduce airway hyperresponsiveness in mice [49]. Further, the study suggested that the response was associated with a guanosine-3,5-monophosphate (cGMP) mechanism both in the presence and absence of airway inflammation. Moreover, Song et al. (2002) suggested that CO could induce significant antiproliferative effects for human airway smooth muscle cells [50]. Thus, further studies are needed to more clearly discern the mechanism underlying the exacerbation of asthma by CO.

This study features three distinct strengths that increase the significance of the observations reported here. First, this is the first study to explore the association between gaseous pollutants and EADs for asthma in the central Sichuan Basin of southwestern China. Although the Bejing–Sichuan belt is located in one of two severe air pollution belts of China [12], investigations of pollution and pollution risks have lagged in the Sichuan Basin due to the relatively depressed economic conditions in the region [13]. The recent improvement and establishment of monitoring systems in the Sichuan Basin have provided opportunities to now conduct such investigations. In addition, several factors render the area a unique research resource that can help fill knowledge-gaps concerning pollution risks in the region. These include the uniquely dense population, different sources of air pollutants, different diffusional dilution conditions, and significantly different economic levels in the Sichuan Basin compared to eastern areas and cities. Second, emergency ambulance dispatch data for the whole city were used in our study due to the unique advantage of the Chengdu First-Aid Command System. City-wide data such as these have been rarely used in previous studies. Third, the city of Chengdu is large and densely populated, which is advantageous for assessing the effects of air pollutants on disease incidence.

However, some limitations were also evident in this study. First, concentrations of air pollutants were obtained from fixed municipal environmental monitoring centers, and thus, individual exposure data were not evaluated. Second, this study was conducted for the central Sichuan Basin. The specific geographical features and weather conditions of the region should be considered, and generalizations of these results should be treated cautiously. Third, not all asthma patients call for emergency ambulances when disease states occur. Consequently, we conducted a time-stratified case-crossover analysis based on individuals from which cumulative effects could not be obtained.

## Conclusions

This study provides important data to advance our limited understanding of potential health risks due to ambient gaseous pollutants. Importantly, these data suggest that increased concentrations of SO_2_, NO_2_, and CO were significantly and positively associated with emergency ambulance dispatches for asthma in Chengdu, China. Further studies are needed to assess the effects of various individual air pollutants on asthma.

## Abbreviations

AP: Atmospheric pressure
CI: Confidence interval
CO: Carbon monoxide
EADs: Emergency ambulance dispatches
IQR: Interquartile range
NO_2_: Nitrogen dioxide
O_3_: Ozone
O_3_-8 h: Daily 8-h mean concentration for O_3_
OR: Odds ratio
PM_2.5_: Particulate matter less than 2.5 μm in aerodynamic diameter
SD: Standard deviation
SO_2_: Sulfur dioxide
tpd: Times per day
